# Aberrant septin 11 is associated with sporadic frontotemporal lobar degeneration

**DOI:** 10.1186/1750-1326-6-82

**Published:** 2011-11-29

**Authors:** Yair M Gozal, Nicholas T Seyfried, Marla Gearing, Jonathan D Glass, Craig J Heilman, Joanne Wuu, Duc M Duong, Dongmei Cheng, Qiangwei Xia, Howard D Rees, Jason J Fritz, Deborah S Cooper, Junmin Peng, Allan I Levey, James J Lah

**Affiliations:** 1 Department of Neurology, Emory University School of Medicine, Atlanta, GA 30322. USA; 2 Department of Human Genetics, Emory University School of Medicine, Atlanta, GA 30322. USA; 3 Emory Proteomics Service Center, Emory University School of Medicine, Atlanta, GA 30322. USA; 4 Center for Neurodegenerative Diseases, Alzheimer's Disease Research Center, Emory University School of Medicine, Atlanta, GA 30322. USA

**Keywords:** Neurodegeneration, dementia, proteomics, mass spectrometry, ubiquitin, aggregates

## Abstract

**Background:**

Detergent-insoluble protein accumulation and aggregation in the brain is one of the pathological hallmarks of neurodegenerative diseases. Here, we describe the identification of septin 11 (SEPT11), an enriched component of detergent-resistant fractions in frontotemporal lobar degeneration with ubiquitin-immunoreactive inclusions (FTLD-U), using large-scale unbiased proteomics approaches.

**Results:**

We developed and applied orthogonal quantitative proteomic strategies for the unbiased identification of disease-associated proteins in FTLD-U. Using these approaches, we proteomically profiled detergent-insoluble protein extracts prepared from frontal cortex of FTLD-U cases, unaffected controls, or neurologic controls (i.e. Alzheimer's disease; AD). Among the proteins altered specifically in FTLD-U, we identified TAR DNA binding protein-43 (TDP-43), a known component of ubiquitinated inclusions. Moreover, we identified additional proteins enriched in detergent-resistant fractions in FTLD-U, and characterized one of them, SEPT11, in detail. Using independent highly sensitive targeted proteomics approaches, we confirmed the enrichment of SEPT11 in FTLD-U extracts. We further showed that SEPT11 is proteolytically cleaved into N-terminal fragments and, in addition to its prominent glial localization in normal brain, accumulates in thread-like pathology in affected cortex of FTLD-U patients.

**Conclusions:**

The proteomic discovery of insoluble SEPT11 accumulation in FTLD-U, along with novel pathological associations, highlights a role for this cytoskeleton-associated protein in the pathogenesis of this complex disorder.

## Background

Frontotemporal lobar degeneration (FTLD) encompasses a heterogeneous group of sporadic and familial diseases associated with circumscribed degeneration of the prefrontal and anterior temporal lobes. As the third most common neurodegenerative cause of dementia behind Alzheimer's disease (AD) and Lewy body dementia, FTLD accounts for 5% of dementia cases irrespective of age [1]. Pathologically, FTLD is equally heterogeneous, and may present as a tauopathy, or more commonly, with tau-negative, ubiquitin-immunoreactive neurites and inclusions [2]. In these cases, termed FTLD-ubiquitin (FTLD-U), histopathology is primarily observed within the small layer II neurons of the frontal and temporal cortices, as well as in granule cells of the dentate gyrus of the hippocampus [3].

In recent years, significant progress has been made in understanding the genetic and neuropathologic basis of FTLD-U. In 2006, mutations in the progranulin gene (*GRN*) were identified as the cause of chromosome 17-linked FTLD-U [4,5]. This discovery was followed by the identification of TAR DNA-binding protein 43 (TDP-43), the first major non-ubiquitin protein component of pathological inclusions in familial and sporadic FTLD-U [6]. Although in normal neurons TDP-43 is a nuclear RNA-binding protein, in pathologic conditions TDP-43 redistributes from the nucleus to the cytoplasm where it is aggregated, phosphorylated, ubiquitinated, and proteolytically cleaved into C-terminal fragments [6]. Notably, TDP-43 is also localized in the intracytoplasmic ubiquitinated inclusions of sporadic amyotrophic lateral sclerosis (ALS), a motor neuron disease often associated with FTLD-U [3,6], and mutations in TDP-43 have been linked to ALS [7-10].

The molecular pathways underlying TDP-43 aggregation and toxicity have not yet been fully elucidated. Fragmentation of TDP-43, possibly by caspase-dependent cleavage [11], and its subsequent cytoplasmic sequestration have been posited as critical factors in promoting cellular toxicity [12,13]. However, several reports have questioned the specificity of the association between TDP-43 and FTLD-U/ALS after TDP-43 immunoreactive aggregates were found in a range of neurodegenerative disorders, including AD and Parkinson's disease (PD) [14,15]. In addition, extensive histopathological characterization of FTLD-U cases with TDP-43 specific antibodies has revealed at least four pathologic FTLD-U subtypes that differ in aggregate distribution, density, and morphology, suggesting that they may not share a common molecular basis [16,17]. Finally, cases caused by mutations in the *CHMP2B *gene, as well as sporadic cases with FUS-immunoreactive inclusions, feature ubiquitin-positive inclusions that lack TDP-43 immunoreactivity [18,19]. Taken together, these findings suggest that other, as yet unknown, proteins may contribute in the pathogenesis of this complex disorder. Thus, in the current study we applied quantitative proteomics methodologies to identify molecular substrates and pathways involved in FTLD-U pathogenesis. Application of these strategies in performing shotgun proteomic analyses of FTLD-U samples resulted in the identification of SEPT11, a novel FTLD-U-associated protein that is enriched in detergent-insoluble fractions and accumulates in the brain of FTLD-U cases.

## Results and Discussion

### Discovery of altered proteins in FTLD-U by LC-MS/MS

**Figure 1:**
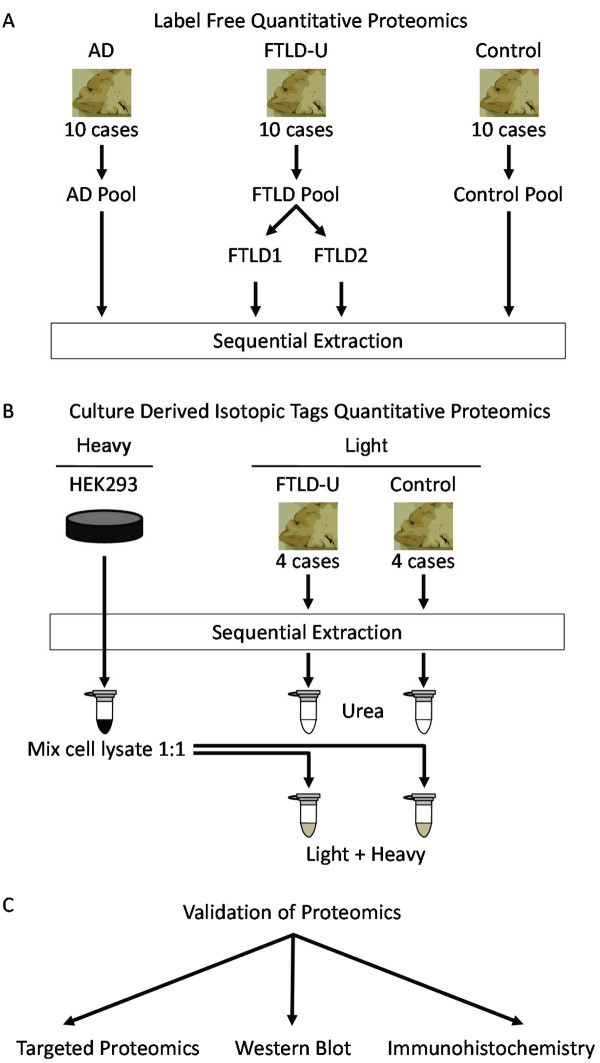
**Diagram of experimental workflow**. (a) Sample preparation for label-free quantitative proteomics (Discovery Phase A). Ten cases each of AD, FTLD-U, and Control were pooled by diagnosis and sequentially extracted. Urea samples were then analyzed by shotgun LC-MS/MS. (b) Sample preparation for quantitative proteomics based on culture derived isotopic tags (Discovery Phase B). Four cases each of FTLD-U and control were pooled by diagnosis and sequentially extracted. Urea fractions for each diagnosis were mixed (1:1) with HEK293 lysate labeled with heavy stable isotopes of arginine and lysine. The heavy labeled peptides served as internal standards following analysis by LC-MS/MS. (c) Validation of identification of Septin 11 enrichment in FTLD-U using three independent methods.

To identify differentially expressed proteins in FTLD-U, which like TDP-43 are enriched in detergent-insoluble brain fractions, we examined post-mortem samples using two independent shotgun quantitative proteomic approaches (Figure 1a-b). In the first strategy, frontal cortex homogenates from 10 cases each of FTLD-U, AD, and control (Table 1) were pooled by diagnosis and serially extracted with buffers of increasing stringency. To allow estimation of the variances associated with sample preparation and analysis, the FTLD-U pooled homogenates were divided into two identical samples and processed in parallel as technical replicates. The resulting four detergent-insoluble, urea-soluble fractions were first separated by SDS-PAGE (Additional File 1a), excised in 5 bands from the gel, trypsin digested, and analyzed via liquid chromatography coupled with tandem mass spectrometry (LC-MS/MS) on a high resolution mass spectrometer. For each of the 512 identified proteins, a quantitative protein comparison based on the extracted ion current (XIC) of individual peptides was performed, and abundance ratios calculated (e.g. FTLD-U/Control). To statistically evaluate and filter the quantitative data, the abundance ratios from all pair-wise comparisons were logarithmically transformed and the resulting histogram of all values was fitted to a Gaussian distribution (Additional Files 2 and 3). In all, 50 proteins were found to be significantly altered in FTLD-U when compared to control and AD extracts.

**Table 1:** Detailed Demographics of Cases Selected for Proteomics

Diagnosis	Case #		^**1**^**PMI (hr)**	Age at Death	Duration	ApoE Status	^**2**^**Race/Sex**
	1		4	55	6	E3/3	W/M
	2		4.5	55	3	E3/4	W/F
	3		20	57	7	E3/4	B/F
	4		11.5	62	10	E3/4	W/M
AD	5		4.5	64	12	E3/4	W/F
	6		5.5	69	21	E4/4	W/F
	7		15	71	2.5	E3/4	W/M
	8		17	71	8	E3/4	W/M
	9		21	76	12	E2/3	W/F
	10		12	81	12	E3/4	W/F

**n = 10**	**AVG**		**11.5**	**66.1**	**9.4**		

	1		3	52		E3/4	W/F
	2		10	57		E3/3	W/M
	3	^3^*	8	60		E3/4	B/F
	4		12	61		E3/4	B/M
Control	5		12	65	NA	E3/3	W/M
	6		6	65		E3/3	W/F
	7	*	11	68		E3/3	W/F
	8	*	6	69		E3/3	W/M
	9		7	74		E3/3	W/F
	10	*	6	75		E3/3	W/F

**n = 10**	**AVG**		**8.1**	**64.6**	**NA**		

	1		3	41	9	E3/3	W/M
	2		17.5	61	5	E3/3	W/M
	3	*	11.5	62	10	E3/3	A/F
	4	*	17	63	7.5	E3/3	W/M
FTLD-U	5		6	64	8	E3/4	W/F
	6	*	NA	69	1	E3/3	W/F
	7		NA	69	1	E3/3	W/F
	8		18	71	9	E3/3	W/F
	9		17	74	16	E3/4	W/M
	10	*	7	83	4	E3/3	W/F

**n = 10**	**AVG**		**12.1**	**65.7**	**7.1**		

^1^Post-mortem interval ^2^**W**hite, **B**lack, **A**sian, **F**emale, **M**ale ^3^Cases marked with asterisk were selected for pooling in CDIT proteomics.

To reduce the variances in the proteomics analysis, we performed an independent study using a separate MS strategy based on the use of culture-derived isotopic tags (CDIT), and cross-referenced the findings with the first label-free approach [20]. In this second approach, whole cell lysate from isotopically-labeled HEK 293 cells was mixed equally with urea-soluble fractions prepared from pools of 4 FTLD-U or 4 control cases (Table 1 marked by asterisk). The heavy-labeled peptides from the cell lysate are chemically identical to their unlabeled counterparts in each urea fraction, and can therefore serve as internal standards for the measurement of protein abundance across samples [21]. The urea/HEK 293 lysate mixtures were subsequently separated by SDS-PAGE (Additional File 1b) and also excised in 5 bands for LC-MS/MS analysis. Using the heavy-labeled internal standard as a reference between FTLD-U and control samples, abundance ratios were calculated, converted into log_2 _values, plotted as a histogram of all proteins, and fitted to a Gaussian distribution (Additional File 4). Of the 1304 proteins identified in this approach, 194 were found to be altered in FTLD-U compared with control. Among the 10 proteins significantly altered in both proteomic discovery sets (Table 2), we identified Septin 11 (SEPT11), a 429 amino acid cytoskeletal GTPase thought to play a role in filament formation [22]. SEPT11 was consistently enriched ~4-fold in FTLD-U compared to control across all molecular weight regions in both proteomics experiments. In the label-free approach, we identified 3 SEPT11 peptides (Additional File 5), and quantification revealed a total signal to noise ratio (SN) of 19.9 in both FTLD-U replicates compared with 4.6 in the control sample. Similarly, we identified the same 3 peptides in the CDIT strategy, as well as an additional peptide, with total SN of 134.4 in FTLD-U and 43.4 in control. As expected, TDP-43 was also increased in FTLD-U, albeit more modestly than SEPT11, showing ~1.8-fold and ~1.3-fold enrichment in the label-free and CDIT quantitative proteomics approaches, respectively. The specificity of our proteomics strategy was further validated by comparison with AD cases, as SEPT11 was not correspondingly increased in insoluble material from AD frontal cortex. This finding indicates that the enrichment of SEPT11 in FTLD-U is not a non-specific association with protein aggregates, and that it is distinct from proteins associated with neurofibrillary tangles and senile plaques. Moreover, known AD-associated proteins, including β-amyloid, tau, and apolipoprotein E, were all enriched in insoluble fractions in AD cases but not in FTLD-U [23].

**Table 2:** Proteins specifically altered in FTLD-U urea fractions using two independent proteomics strategies.

	GeneBank™	Label-Free Approach		CDIT Approach
Protein	Accession Number	**mean Log**_**2 **_**Ratio**		**Log**_**2 **_**Ratio**
		FTLD-U/Control	FTLD-U/AD	FTLD-U/Control
Septin 11	NP_060713.1	2.0	2.4	1.8
Excitatory Amino Acid Transporter 2	NP_004162.2	2.0	1.5	4.2
Glial Fibrillary Acidic Protein	NP_002046.1	1.9	1.3	2.2
Alpha Catenin	NP_001894.2	1.3	1.3	3.9
Aquaporin 4	NP_004019.1	1.1	0.9	10.1
Protein Phosphatase 2	NP_055040.2	1.0	1.2	1.8
Transketolase	NP_001055.1	0.9	1.1	2.2

2,4-Dienoyl CoA Reductase 2	NP_065715.1	-1.1	-0.9	-3.2
Rho GTPase Activating Protein 1	NP_004299.1	-1.5	-1.0	-1.6
Synaptosomal-Associated Protein 25	NP_003072.2	-1.5	-1.0	-4.9

### Validation of SEPT11 enrichment in FTLD-U

**Figure 2:**
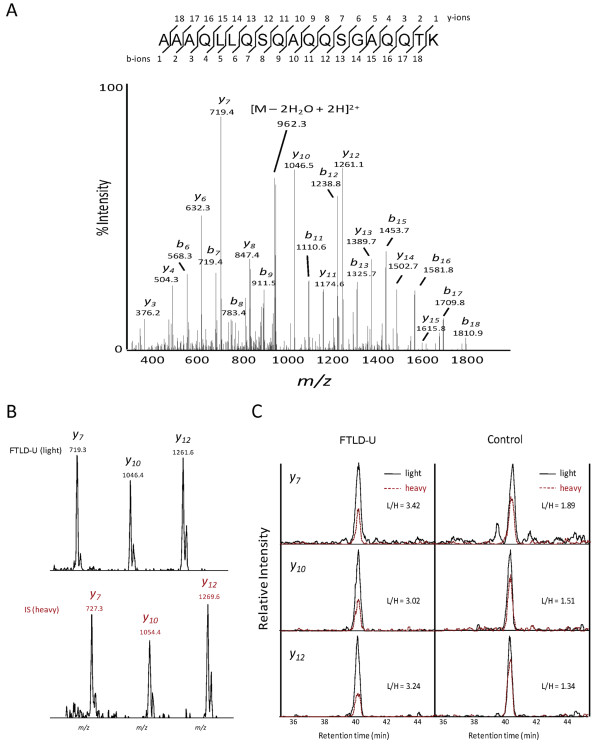
**Targeted proteomics to quantify SEPT11 enrichment in urea fractions**. (a) MS/MS scan of the precursor ion (*m/z *979.0) corresponding to a unique SEPT11 peptide (amino acids 400-418). (b) MS/MS product ions selected for quantitation in light (top) and heavy (bottom) peptide forms. (c) Extracted ion chromatogram of targeted spectra for each of the product ions in (b). The spiked heavy- (red) and endogenous light (black) peptides have identical retention times and are overlaid. For each product ion, enrichment is calculated as a ratio of light to heavy signal of the area under each curve.

Septins are a highly conserved family of filamentous proteins first identified in yeast as a group of cell division cycle regulatory genes [24]. The 14 mammalian septins identified to date share a central characteristic P-loop GTP-binding domain flanked N- and C-termini which vary in length and amino acid composition [25]. Based on their amino acid sequence homology and their capacity to interchange with other septins in heteromeric complexes that underlie filament assembly, mammalian septins can be further classified into four subgroups. SEPT11 belongs to the septin 6 (SEPT6) subfamily, which along with other subgroup members (septins 8 and 10) may provide for redundancy in the formation of the septin 2/6/7 and 7/9b/11 filamentous complexes [22,26]. SEPT11 and SEPT6 share 85% homology, and both are expressed in the CNS [27-29]. Therefore, to confirm the proteomic identification of SEPT11 in FTLD-U urea fractions, we first used a targeted mass spectrometry approach to map unique SEPT11 peptides in SEPT11-overexpressing HEK 293 cells. We subsequently selected 5 of these unique peptides and validated their presence in urea fractions prepared from 4 pooled FTLD-U cases (Additional File 6). Finally, we re-quantified SEPT11 at its expected molecular weight (Gel piece 3; 40-60 kDa) in the pooled urea samples containing the heavy-labeled SEPT11 HEK 293 cell lysate. Specifically, we monitored multiple high intensity product ions, which serve as surrogates for measurement of peptide intensities, from two unique peptides corresponding to amino acids 66-79 and 400-418 (Figure 2). SEPT11 was enriched 2.1-fold (± 0.2) in FTLD-U compared to control in this gel band (Table 3). In contrast, concurrent examination of 3 β-actin peptides revealed a slight reduction of this reference protein in FTLD-U samples (0.7 ± 0.1).

**Table 3:** Targeted MS conditions for Septin11 peptides in gel band corresponding to 40-60 kDa.

		^**1**^**Precursor Ions (*m/z*)**		^**5**^**Product Ions (*m/z*)**			^**6**^**AUC (FTLD-U)**		AUC (Control)		FTLD-U/Control
Protein Name	Peptide Sequences										
		**Native (**^**2**^**L)**	^**3**^**IS **(^**4**^**H)**	Name	Native (L)	IS (H)	(L)	(H)	(L)	(H)	
Septin11	FESDPATHNEPGVR	778.4	783.4								
				y2-10	539.4	544.4	2420	1034	1776	1531	2.0
				y1-10	1077.3	1087.3	5428	1176	3548	1797	2.3
Septin11	AAAQLLQSQAQQSGAQQTK	979.0	983.0								
				y1-7	719.3	727.3	2700	790	1747	921	1.8
				y1-10	1046.4	1054.4	2242	743	1559	1030	2.0
				y1-12	1261.6	1269.6	3476	1074	1790	1334	2.4

											**2.1 ± 0.2**
		**Precursor Ions (*m/z*)**					^**7**^**XIC (FTLD-U)**		**XIC (Control)**		
							
		**Native (L)**	**IS (H)**				**(L)**	**(H)**	**(L)**	**(H)**	

β-actin	AGFAGDDAPR	488.73	493.74				3208	916	3799	717	0.7
β-actin	GYSFTTTAER	566.77	571.78				2651	740	4109	896	0.8
β-actin	SYELPDGQVITIGNER	895.96	900.96				2474	704	2806	584	0.7

											**0.7 ± 0.1**

^1^The m/z values of native and labeled peptides ^2^Light ^3^Internal Standard ^4^Heavy ^5^The name and m/z values of monitored product ions (e.g. y1-7, the singly-charged y7 ion) ^6^Area under the curve ^7^Extracted ion chromatogram.

**Figure 3:**
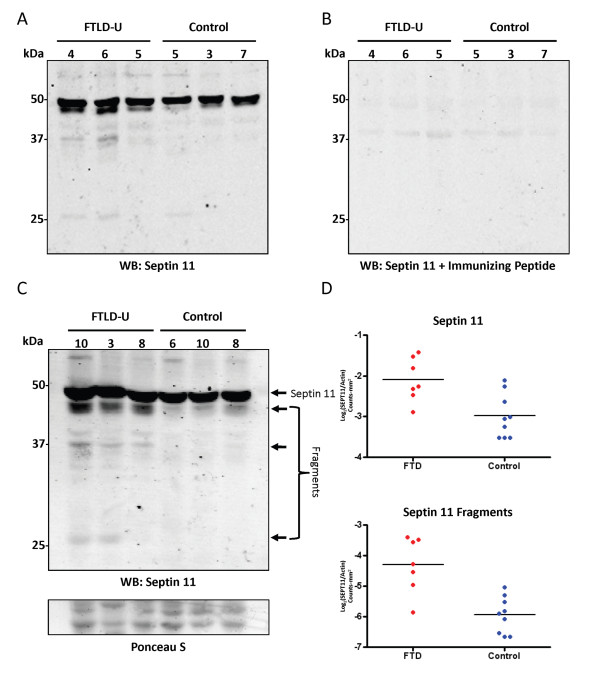
**Immunoblots of SEPT11 confirm proteomic findings**. (a) Frontal cortex samples from individual FTLD-U and control cases were sequentially extracted with buffers containing triton X-100, sarkosyl, and urea. Urea fractions were immunoblotted with an affinity purified SEPT11 rabbit polyclonal antibody raised to a peptide at the extreme N-terminus. (b) Preabsorption using the immunizing peptides quenches immunoreactivity with the exception of a faint non-specific band at ~40 kDa. (c) Overexposed immunoblot of urea fractions from additional FTLD-U and control cases detects SEPT11 at its expected M_r _(49 kDa), as well as additional N-terminal species at ~45 kDa, ~37 kDa, and ~28 kDa. (D) Quantification in 7 FTLD-U cases and 9 controls of immunoblot band intensities for 49 kDa band (top panel) and all 3 SEPT11 lower M_r _fragments (bottom panel). The band at ~40 kDa that did not preabsorb in (b) was excluded from the analysis.

The 2-fold discrepancy in SEPT11 enrichment obtained via the shotgun proteomics analyses and the highly sensitive and specific targeted proteomics strategy, suggests that SEPT11 may also be enriched in additional molecular weight regions. Stratification of the data obtained in the proteomic approach using CDIT by molecular weight demonstrated widespread distribution of SEPT11 peptides throughout the entire mass range, with spectra identified in every gel band. Quantitation using XIC and the heavy-labeled internal standard showed a 1.6 to 2-fold enrichment of SEPT11 at the 40-60 kDa range, with higher and lower molecular weight regions accounting for the remainder of the ~4-fold change in these samples. To confirm the changes observed by mass spectrometry, we performed immunoblots on FTLD-U and control urea fractions using a rabbit polyclonal antibody raised to a peptide (amino acids 1-15) at the extreme N-terminus of SEPT11 (Figure 3). In addition to the expected band at 49 kDa corresponding to the full length protein, we detected 3 specific SEPT11-immunoreactive bands at ~45 kDa, ~37 kDa, and ~28 kDa in FTLD-U urea fractions that were absent or reduced in control extracts (Figure 3c) and in the corresponding detergent-soluble fractions (Additional File 7). Quantification of immunoblot band intensities showed a modest but significant 1.8-fold enrichment (*p *= 0.006) of the full-length SEPT11 band in frontal cortex urea fractions from 7 FTLD-U and 9 control cases (Figure 3d), supporting the quantitative mass spectrometric measurements at this molecular weight. Moreover, SEPT11 lower M_r _fragments were 3.1-fold enriched in these FTLD-U cases compared to controls (*p *< 0.001). The proteolytic cleavage of SEPT11 is consistent with that observed for other pathologic aggregating proteins, including TDP-43 and tau, where polymerization, post-translational modification, and degradation underlie protein insolubility [6,30].

**Figure 4:**
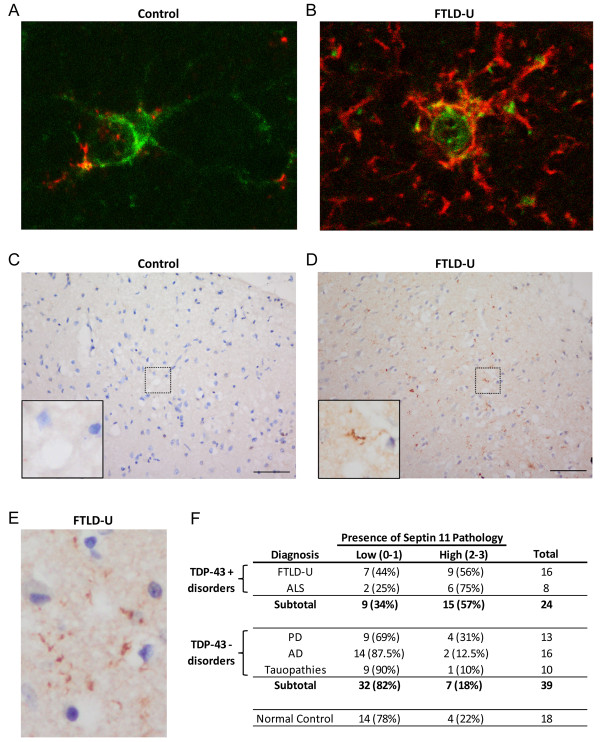
**SEPT11 immunoreactivity in control and FTLD-U using polyclonal N-terminal antibody**. (a-b) Double label immunofluorescence in control (a) and FTLD-U (b) frontal cortex reveals overlap between SEPT11 (red) and glial fibrillary acidic protein (GFAP; green), suggesting that cell-associated staining localizes to astrocyte cell bodies and processes. (c-e) IHC in FTLD-U (d and e) frontal cortex detects both linear and rounded accumulations of SEPT11 that are absent in control (c). Unlike in (a) and (b), these SEPT11 threads were not associated with specific cell bodies. (f) Summary of blinded scoring of frontal cortex from 81 cases and controls for the presence of SEPT11 threads.

To examine if the SEPT11 biochemical insolubility reflects an altered distribution in FTLD-U, we performed immunohistochemical analysis using our specific N-terminal polyclonal antibody. SEPT11 immunoreactivity was localized with astrocytic cell bodies and processes in frontal cortex of FTLD-U and control cases (Figure 4a-b). Moreover, we observed neuropil immunoreactivity consistent with the localization of SEPT11 along dendrites and in post-synaptic membranes as has been previously described [31]. Analysis revealed no SEPT11 overlap with TDP-43 and ubiquitin-positive neuronal inclusions. However, in a subset of FTLD-U cases, we identified numerous fibrillar thread-like structures which did not associate with glial processes or nuclei (Figure 4c-e). These SEPT11-immunoreactive features localized primarily in superficial cortical layers (layers 2-3), and appeared irregular in shape, length, and thickness. Antibody specificity was confirmed using peptide competition studies, resulting in the complete loss of immunoreactivity in glia, neuropil, and pathologic threads. In contrast, we were unable to preadsorb a thread-like pathology seen with a separate commercially available SEPT11 antibody.

To determine whether SEPT11 threads correspond to a disease-specific signature, we examined and scored this characteristic staining in a series of FTLD-U, ALS, neurologic disease control (AD, PD, and tauopathies), and normal control cases in a blinded fashion using a semi-quantitative 4-tiered rating scale (Additional File 8). SEPT11 threads were markedly enriched in FTLD-U, with 56% of cases (9/16) scored as having positive or very positive (score ≥ 2) staining of threads (Figure 4f). In comparison, only 18% (7/39; *p *= 0.008) of neurologic controls and 22% (4/18; *p *= 0.076) of normal controls were scored in this range. Moreover, when FTLD-U and ALS, the two disease groups associated with TDP-43, were examined together and compared to the TDP-43 negative neurologic controls (AD, PD, and tauopathies), the difference in SEPT11 pathology was even stronger (*p *< 0.001). This provided additional support for shared pathogenic mechanisms in FTLD-U and ALS. Since their presence is highly specific to FTLD-U and ALS, SEPT11-immunoreactive threads may reflect pathologic accumulations of the insoluble full-length or fragmented protein identified in urea fractions.

## Conclusions

Perturbed expression of septins, a family of cytoskeletal GTP-binding proteins, has been extensively associated with neurodegeneration. Notably, SEPT1, SEPT2, and SEPT4 have been shown to co-localize with neurofibrillary tangles and dystrophic neurites in AD [32]. In PD, SEPT4 co-localizes with α-synuclein in Lewy bodies [33], and SEPT5 has been shown to be a target for parkin-mediated ubiquitination [34]. In this study, we have shown that SEPT11 is biochemically altered and accumulates in FTLD-U. Although the specific functions of SEPT11 in the brain are not well defined, SEPT11 is thought to form multi-septin complexes that assemble into filaments and function as scaffolds for cytoskeletal-binding proteins [35]. Supporting this potential role, SEPT11 has been shown to colocalize with microtubules and stress fibers in multiple epithelial cell lines [36]. Thus, it is plausible that changes in SEPT11 solubility could disrupt cytoskeletal function and result in cellular toxicity, a mechanism already established for tau-based proteopathies in other neurodegenerative disorders [37]. In fact, SEPT11 insolubility and toxicity may be related to the presence of lower M_r _fragments in FTLD-U urea fractions identified by immunoblotting (Figure 3). This would parallel findings in a highly homologous protein, SEPT6, in which N-terminal fragments containing the variable region and GTP-binding domain have been reported to be insoluble [38]. As is seen with multiple disease proteins, including TDP-43, tau, α-synuclein, and Aβ [39,40], proteolytic cleavage of SEPT11 may result in protein misfolding, aggregation, and cytotoxicity. Finally, truncation of the SEPT11 C-terminus would also impact the coiled-coil domain of the protein, a region critical for septin-septin interactions as well as for binding to non-septin partners [31,38].

Our characterization of SEPT11 in FTLD-U establishes a powerful approach to large-scale identification of disease-associated proteins in neurodegenerative conditions through analysis of the detergent-insoluble proteome. SEPT11 immunoreactive threads are a novel neuropathological feature of a subgroup of FTLD-U cases, but accumulation of insoluble SEPT11 aggregates is not a universal feature of all FTLD-U cases. Our proteomics-based strategy incorporated pooled samples of FTLD-U and control cases to reduce the impact of biological variability that is unavoidable and uncontrollable in studies of human autopsy materials. Factors, such as postmortem interval, unknown agonal circumstances, and other individual features unique to every patient, introduce enormous noise in efforts to identify disease-specific elements. Although accepted criteria for FTLD-U have been established, our understanding of this neuropathological entity is far from complete. This is clearly demonstrated by ongoing efforts to subclassify FTLD-U cases based on specific features of TDP-43 immunoreactivity. We have now shown that pathological accumulation of SEPT11 in superficial cortical layers defines an additional molecular feature linked to FTLD-U, and closer examination of candidates identified through proteomic profiling will reveal additional features that will produce a clearer understanding of this complex neurodegenerative disorder.

## Methods

### Case Material

Post-mortem human brain tissues corresponding to diagnoses of FTLD-U, AD, PD, ALS, tauopathy, and unaffected control were obtained from the Alzheimer's Disease Research Center (ADRC) and Center for Neurodegenerative Disease (CND) Brain Bank at Emory University School of Medicine. Diagnoses of FTLD-U, AD, and control cases were made in accordance with established criteria as described previously [41,42]. Diagnoses of PD/Lewy Body Dementia [43], ALS [44], and tauopathy [45-47] were also made based on established criteria.

### Antibodies

In-house SEPT11 antibody (rabbit polyclonal) was raised against keyhole limpet hemocyanin (KLH)-conjugated peptide AVAVGRPSNEELRN at the extreme N-terminus of the protein. Serum from the immunized rabbit was tested by immunoblot and affinity-purified on a column using the immunizing peptide. Commercially available primary antibodies used in these studies were septin 11 (N-15; Santa Cruz Biotechnology, Santa Cruz CA) and glial fibrillary acidic protein (DAKO, Carpinteria CA).

### DNA Constructs

The full-length SEPT11 coding sequence (nucleotides 1-1287, encoding amino acids 1-429) was amplified by PCR from a commercial expression plasmid (Origene, Rockville MD; Clone ID TC113610) using forward primer 5'-AGCTGCGTCTAGAATGGCCGTGGCCGT GGG-3' and reverse primer 5'-ATACGATATTTCTAGAACTGCAAAAGCAGGTGAATG-3'. The PCR products were digested and cloned into the pcDNA3.1-HA expression vector downstream of the two-HA motifs (peptide YPYDVDYA). The N-terminal HA expression vectors were confirmed by DNA sequencing (Agencourt Bioscience Corporation, Beverly MA).

### Sequential Biochemical Fractionation

Sequential extractions were performed as previously described [23] with buffers containing 1% Triton X-100, 1% N-Lauroyl-sarcosine (sarkosyl), and finally 7M urea/2M thiourea. Generally, the protein yield in urea samples during sequential extraction was approximately 2% of the starting material.

### Protein Identification and Quantification by LC-MS/MS

XIC-based label-free quantitative proteomics on pooled urea-soluble fractions was performed as previously described [23]. For quantitative proteomics with CDIT, HEK293 cells were cultured in DMEM (deficient in L-Lysine and L-Arginine) supplemented with 2% dialyzed fetal calf serum (Invitrogen) as described [48]. For stable isotopic labeling, heavy forms L-Arginine and L-Lysine were added (Arg10/Lys8, Cambridge Isotope Laboratories) to a final concentration of 0.13 mM. Excess proline was added at 200 mg/L to block the conversion of arginine to proline. Prior to SDS PAGE, 10 μg of heavy labeled HEK whole cell lysate was added to 10 μg of unlabeled urea fraction from 4 pooled FTLD-U or control cases. The mixed (light and heavy) samples were reduced with 10 mM DTT, and resolved on a 10% polyacrylamide SDS gel. After staining with Coomassie blue, each gel lane was cut into five gel bands, and bands were subjected to in-gel digestion (12.5 μg/ml trypsin). Extracted peptides were analyzed via LC-MS/MS as described previously [23]. To compare medium and heavy labeled samples, database searches were modified to include static modifications of +4.02511 on Lys and +6.02013 on Arg, and dynamic modifications of +3.98909 on Lys and +3.98814 on Arg to account for mass difference between medium and heavy labeled peptides. Quantitative pair-wise comparisons between FTLD-U and control using the internal standard were carried out as described previously [21,31,49].

### Quantitative analysis of SEPT11 by targeted proteomics

For targeted proteomics and peptide mapping of SEPT11, metabolically heavy-labeled HEK 293 cells were transfected with tagged HA-SEPT11 constructs and harvested. The cell lysate alone (for peptide mapping) or mixed with urea extracts from 4 pooled FTLD-U cases was analyzed by LC-MS/MS in a data-independent MS/MS mode to specifically identify and fragment ions corresponding to five pre-selected SEPT11 peptides (Additional File 6). Quantitative analysis was performed with non-transfected heavy-labeled HEK 293 cell lysate as internal standards. The labeled cells were spiked into detergent-insoluble fractions from 4 pooled FTLD-U and 4 pooled control cases. Samples were then resolved by SDS-PAGE, and the gel piece corresponding to 40-60 kDa in each sample was subjected to trypsin in-gel digestion. We then specifically quantified MS/MS ion pairs for SEPT11 peptides AAAQLLQSQAQQSGAQQTK and FESDPATHNEPGVR.

### Immunohistochemistry

Paraffin-embedded sections of hippocampus and frontal cortex (8 μm thick) were deparaffinized and microwaved in citrate buffer (0.01 M, pH 6) for 10 minutes. After cooling to room temperature for 1 hour, sections were rinsed and endogenous peroxidase activity was blocked with 3% hydrogen peroxide at 40°C. Sections were then incubated with normal goat serum for 15 minutes at 40°C, followed by primary antibody (diluted in 1% BSA) overnight at 4°C. The following day, sections were incubated with biotinylated goat secondary antibody for 30 minutes at 37°C, and finally with avidin-biotin peroxidase complex (Vector Laboratories) for 60 minutes at 37°C. 3,3'-Diaminobenzidine (DAB) was used as the chromogen for color development and was followed with hematoxylin counterstain.

### Scoring of SEPT11 Immunoreactivity

Scoring of specific SEPT11 immunoreactivity in paraffin-embedded sections of frontal cortex was done in a blinded fashion by three independent raters. Scoring was limited to superficial cortical layers (layers 2-3) and included only thread-like structures not directly associated with glial nuclei or processes. Astrocytic SEPT11-staining was specifically excluded and was, therefore, not evaluated. Staining was scored as negative (0; no immunoreactivity), equivocal (1; very few threads), positive (2; threads common throughout superficial layers or showing patchy dense distribution), or very positive (3; threads densely distributed throughout superficial layers). A consensus score was determined for each of the cases examined, and typically corresponded to the median score of the three scorers. In rare cases where the three scorers differed by more than 1 point, cases were re-evaluated in the presence of all three scorers. In analyses, samples scored as negative or equivocal were grouped together as "Low Presence of SEPT11 Threads," while samples scored as positive or very positive were grouped together as "High Presence of SEPT11 Threads." We considered age of onset, age at death, post-mortem interval, disease duration, tissue quality, and tissue volume as potential confounders for statistical analyses. However, none of these parameters was significantly associated with predicting the presence or absence of SEPT11 threads. Thus, statistical comparisons were conducted using Fisher's exact test.

### Western Blotting

Immunoblotting was performed according to standard procedures as described previously [49]. Notably, acquired data were analyzed with GraphPad Prism 4.0 software (La Jolla, CA) and statistical significance was determined by unpaired two-tailed Student's t test with α = 0.05. For preabsorption studies of SEPT11, primary antibodies were preincubated with 100× molar excess of the antigen overnight at 4°C before incubation with the blot.

## List of Abbreviations

FTLD: frontotemporal lobar degeneration; FTLD-U: frontotemporal lobar degeneration with ubiquitinated inclusions; LC-MS/MS: liquid chromatography and tandem mass spectrometry; XIC: extracted ion current; CDIT: culture derived isotopic tags.

## Competing interests

The authors declare that they have no competing interests.

## Authors' contributions

YMG, JP, AIL, and JJL conceived and designed the overall study with help from NTS (proteomics), QX (proteomics), MG (pathology), JDG (pathology), JW (pathology), JJF (DNA constructs), and CJH (immunoassays). YMG, NTS, DMD, DC, QX, and JP performed the proteomics studies. YMG, MG, JDG, HDR, JJF, DSC, AIL, and JJL performed the IHC and immunoblotting experiments. YMG, CJH, AIL, and JJL designed, developed, and characterized the SEPT11-specific antibody. JW carried out statistical analyses in immunohistochemical studies. YMG drafted the manuscript with help from all co-authors. JP, AIL, and JJL coordinated the study and finalized the manuscript. All authors read and approved the final manuscript.

## Supplementary Material

Additional file 1 **Separation of pooled urea samples by SDS-PAGE for proteomic analysis**. (a) SDS-PAGE gel of urea fractions extracted from AD, FTLD-U, and control pooled frontal cortex homogenates (10 cases each). (b) SDS-PAGE gel of 4 pooled FTLD-U or 4 pooled control samples after addition of heavy labeled cell lysate. The gels were stained with Coomassie Blue G-250 and gel lanes were excised in 5 pieces as indicated (F1-F5 or C1-C5). Click here for file

Additional file 2 **Statistical evaluation and filtering of label-free proteomics data**. (a) Abundance ratios for FTLD-U/Control comparison were transformed (logarithmic base 2) and plotted with each point corresponding to the number of proteins in 0.3 unit windows (black line). A Gaussian curve was subsequently fitted to the data (red line) and used to determine significance levels for protein change. (b) Fitted normal distributions for all possible pair-wise comparisons. Statistical means, standard deviations, and regression coefficients are presented in Additional File 3. Click here for file

Additional file 3 **Statistical data for fitted normal distributions**. Table detailing the specific properties of each fitted normal distribution used for proteomic data analysis. Click here for file

Additional file 4 **Statistical evaluation and filtering of CDIT proteomics data**. Abundance ratios for FTLD-U/Control comparison were transformed (logarithmic base 2) and plotted with each point corresponding to the number of proteins in 0.4 unit windows (black line). A Gaussian curve was subsequently fitted to the data (red line) and used to determine significance levels for protein change. Click here for file

Additional file 5 **Peptide map of SEPT11**. Amino acid sequence of full length SEPT11 (429 aa) marked with peptides identified in shotgun proteomics approaches (solid underline), mapped unique peptides in targeted proteomics (red color), peptides quantified using targeted proteomics (bold), and immunizing peptide used in development of in-house SEPT11 rabbit polyclonal antibody (dotted underline). Click here for file

Additional file 6 **SEPT11 peptides selected for targeted proteomics**. Table detailing properties of SEPT11 peptides selected for targeted proteomics. Click here for file

Additional file 7 **Assessment of SEPT11 in detergent-soluble fractions by immunoblotting**. Triton X-100 (a) and Sarkosyl (b) fractions extracted from frontal cortex samples of individual FTLD-U and control cases were immunoblotted with an N-terminus specific rabbit polyclonal SEPT11 antibody. While full-length SEPT11 (49 kDa) is abundant in both detergent-soluble fractions, the low molecular weight fragments noted in the urea fractions (Figure 3) were decreased or absent in these fractions. The band noted at ~40 kDa is non-specific as determined by preabsorption studies using the immunizing peptide. Click here for file

Additional file 8 **Individual Case Demographic and Scoring Information**. Table detailing specific demographic data and scoring results for all cases analyzed by immunohistochemistry. Click here for file
