# Supplementary figures and images for: Aberrant septin 11 is associated with sporadic frontotemporal lobar degeneration
Source: Mol Neurodegener. 2011 Nov 29;6:82. doi: 10.1186/1750-1326-6-82 (PMC3259087; doi:10.1186/1750-1326-6-82)

**a**

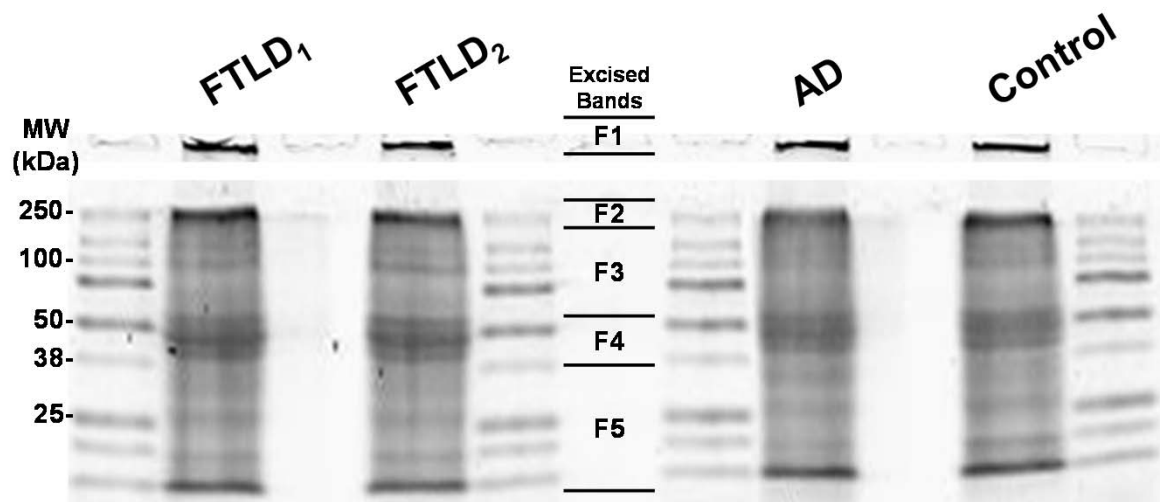

**b**

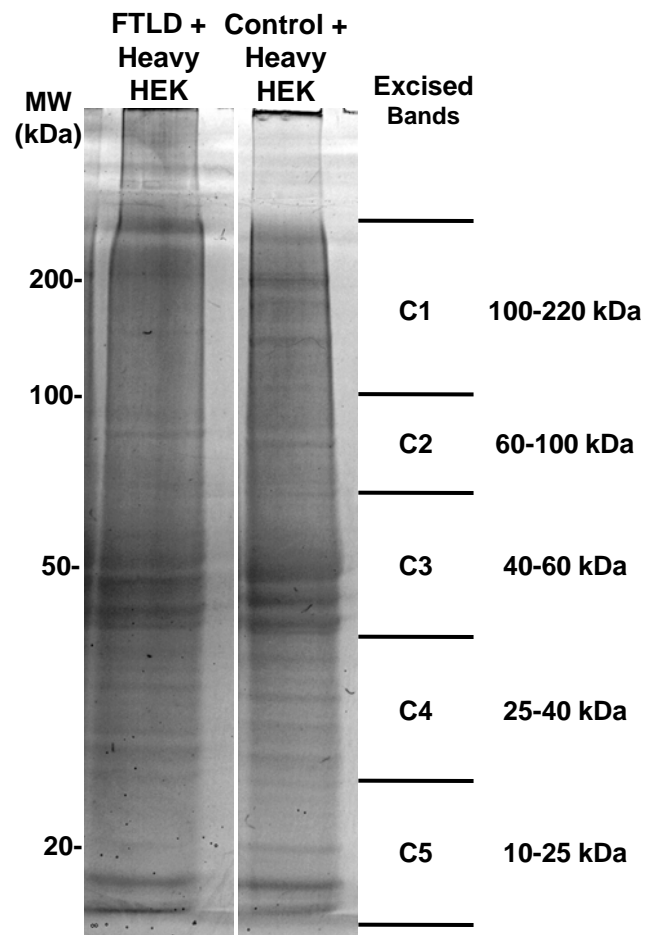

Supplement: Additional file 1 — Separation of pooled urea samples by SDS-PAGE for proteomic analysis. (a) SDS-PAGE gel of urea fractions extracted from AD, FTLD-U, and control pooled frontal cortex homogenates (10 cases each). (b) SDS-PAGE gel of 4 pooled FTLD-U or 4 pooled control samples after addition of heavy labeled cell lysate. The gels were stained with Coomassie Blue G-250 and gel lanes were excised in 5 pieces as indicated (F1-F5 or C1-C5). [file 1750-1326-6-82-S1.PDF]

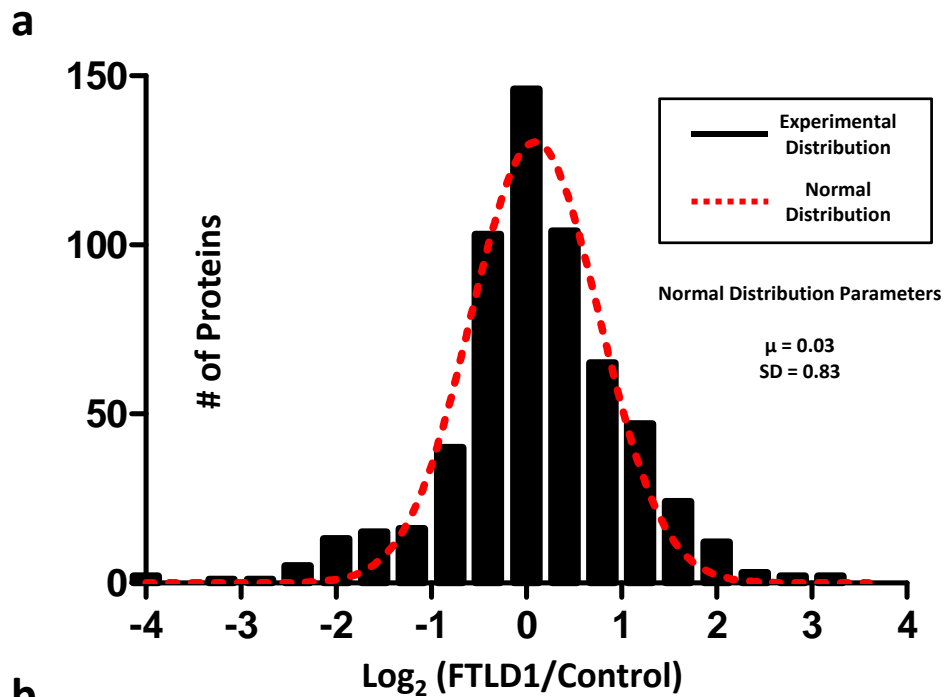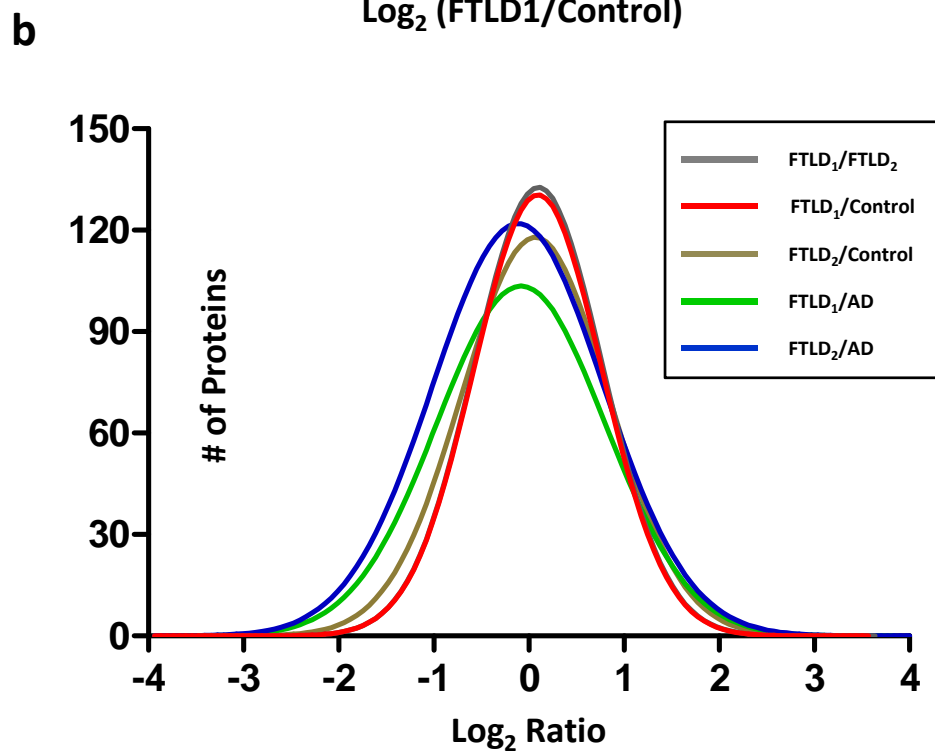

Supplement: Additional file 2 — Statistical evaluation and filtering of label-free proteomics data. (a) Abundance ratios for FTLD-U/Control comparison were transformed (logarithmic base 2) and plotted with each point corresponding to the number of proteins in 0.3 unit windows (black line). A Gaussian curve was subsequently fitted to the data (red line) and used to determine significance levels for protein change. (b) Fitted normal distributions for all possible pair-wise comparisons. Statistical means, standard deviations, and regression coefficients are presented in Additional File 3. [file 1750-1326-6-82-S2.PDF]

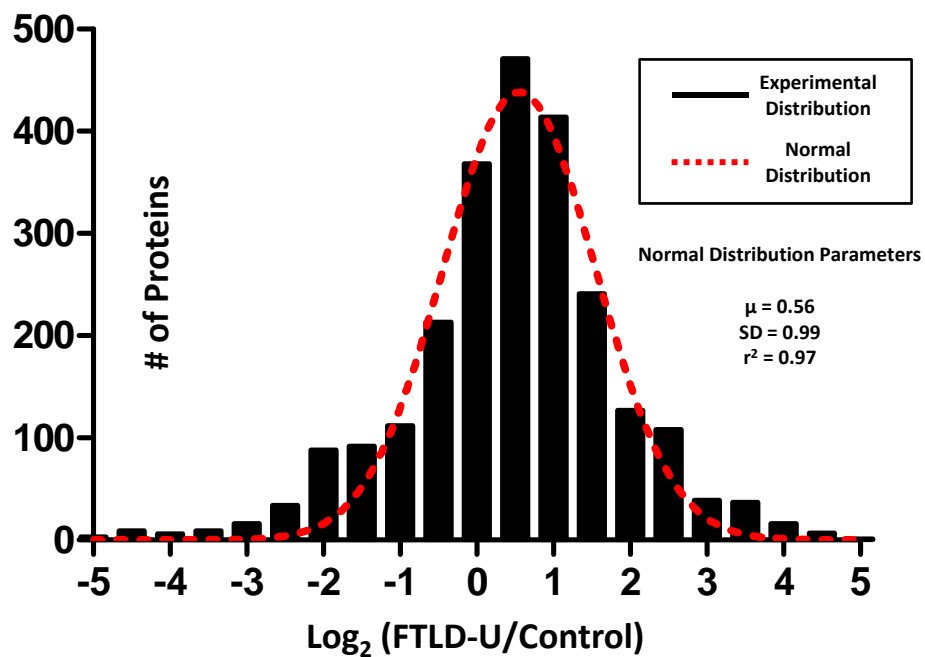

Supplement: Additional file 4 — Statistical evaluation and filtering of CDIT proteomics data. Abundance ratios for FTLD-U/Control comparison were transformed (logarithmic base 2) and plotted with each point corresponding to the number of proteins in 0.4 unit windows (black line). A Gaussian curve was subsequently fitted to the data (red line) and used to determine significance levels for protein change. [file 1750-1326-6-82-S4.PDF]

**A**

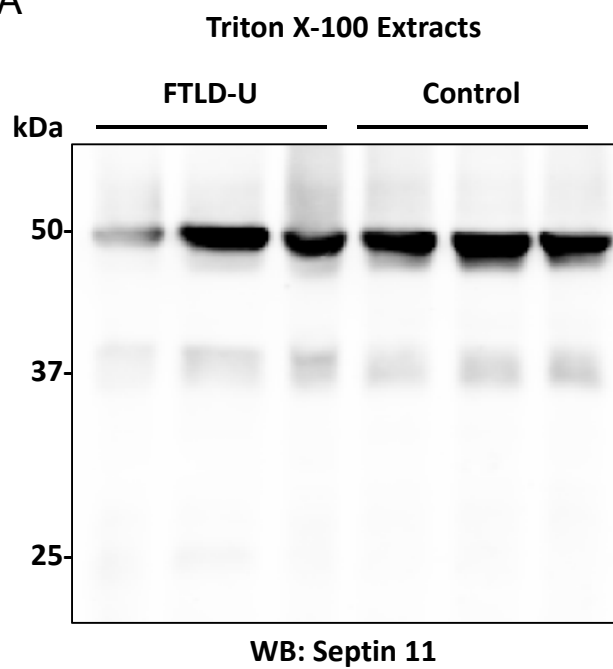

**B**

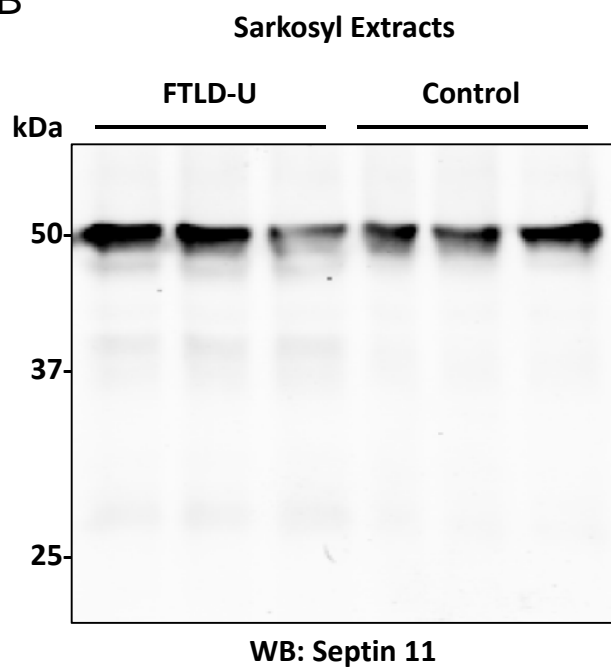

Supplement: Additional file 7 — Assessment of SEPT11 in detergent-soluble fractions by immunoblotting. Triton X-100 (a) and Sarkosyl (b) fractions extracted from frontal cortex samples of individual FTLD-U and control cases were immunoblotted with an N-terminus specific rabbit polyclonal SEPT11 antibody. While full-length SEPT11 (49 kDa) is abundant in both detergent-soluble fractions, the low molecular weight fragments noted in the urea fractions (Figure 3) were decreased or absent in these fractions. The band noted at ~40 kDa is non-specific as determined by preabsorption studies using the immunizing peptide. [file 1750-1326-6-82-S7.PDF]
