# Supplementary material for: Aberrant septin 11 is associated with sporadic frontotemporal lobar degeneration
Source: Mol Neurodegener. 2011 Nov 29;6:82. doi: 10.1186/1750-1326-6-82 (PMC3259087; doi:10.1186/1750-1326-6-82)
Supplement: Additional file 5 — Peptide map of SEPT11. Amino acid sequence of full length SEPT11 (429 aa) marked with peptides identified in shotgun proteomics approaches (solid underline), mapped unique peptides in targeted proteomics (red color), peptides quantified using targeted proteomics (bold), and immunizing peptide used in development of in-house SEPT11 rabbit polyclonal antibody (dotted underline). [file 1750-1326-6-82-S5.PDF]

1- MAVAVGRPSN EELRNLSLSG HVGFDSLPDQ LVNKSTSQGF CFNILCVGET

51 GIGKSTLMDT LFNTK**FESDP ATHNEPGVR**L KARSYELQES NVRLKLTIVD

101 TVGFGDQINK DDSYKPIVEY IDAQFEAYLQ EELKIKRSLF NYHDTRIHAC

151 LYFIAPTGHS LKSLDLVTMK KLDSKVNIIP IIAKADTIAK NELHKFKSKI

201 MSELVSNGVQ IYQFPTDEET VAEINATMSV HLPFAVVGST EEVKIGNKMA

251 KARQYPWGVV QVENENHCDF VKLREMLIRV NMEDLREQTH TRHYELYRRC

301 KLEEMGFKDT DPDSKPFSLQ ETYEAKRNEF LGELQKKEEE MRQMFVMRVK

351 EKEAELKEAE KELHEKFDLL KRTHQEEKKK VEDKKKELEE EVNNFQKKK**A**

401 **AAQLLQSQAQ QSGAQQTK**KD KDKKNASFT -429
